# Faecal shedding and strain diversity of *Listeria monocytogenes *in healthy ruminants and swine in Northern Spain

**DOI:** 10.1186/1746-6148-5-2

**Published:** 2009-01-08

**Authors:** Jon I Esteban, Beatriz Oporto, Gorka Aduriz, Ramón A Juste, Ana Hurtado

**Affiliations:** 1 Department of Animal Health, NEIKER – Instituto Vasco de Investigación y Desarrollo Agrario, Berreaga 1, 48160 Derio, Bizkaia, Spain

## Abstract

**Background:**

*Listeria monocytogenes *is among the most important foodborne bacterial pathogens due to the high mortality rate and severity of the infection. *L. monocytogenes *is a ubiquitous organism occasionally present in the intestinal tract of various animal species and faecal shedding by asymptomatically infected livestock poses a risk for contamination of farm environments and raw food at the pre-harvest stages. The aim of this study was to determine the prevalence and strain diversity of *L. monocytogenes *in healthy ruminants and swine herds.

**Results:**

Faecal samples from 30 animals per herd were collected from 343 herds (120 sheep, 124 beef cattle, 82 dairy cattle and 17 swine) in the Basque Country and screened in pools by an automated enzyme-linked fluorescent immunoassay (VIDAS^®^) to estimate the prevalence of positive herds. Positive samples were subcultured onto the selective and differential agar ALOA and biochemically confirmed. *L. monocytogenes *was isolated from 46.3% of dairy cattle, 30.6% beef cattle and 14.2% sheep herds, but not from swine. Within-herd prevalence investigated by individually analysing 197 sheep and 221 cattle detected 1.5% of faecal shedders in sheep and 21.3% in cattle. Serotyping of 114 isolates identified complex 4b as the most prevalent (84.2%), followed by 1/2a (13.2%), and PFGE analysis of 68 isolates showed a highly diverse *L. monocytogenes *population in ruminant herds.

**Conclusion:**

These results suggested that cattle represent a potentially important reservoir for *L. monocytogenes *in the Basque Country, and highlighted the complexity of pathogen control at the farm level.

## Background

*Listeria monocytogenes *is a ubiquitous organism that is occasionally present in the intestinal tract of various animal species and can cause severe illness in humans after the ingestion of contaminated food products. Although the annual incidence of human listeriosis is quite low in the Basque Country (1.0 cases per 100,000 inhabitants in 2006) compared to salmonellosis (77.2 cases) or campylobacteriosis (114.5 cases) [1], *L. monocytogenes *is among the most important foodborne bacterial pathogens due to the high mortality rate (20–30% mortality) and severity of the disease particularly among pregnant women, neonates and immunosuppressed adults. In addition, *L. monocytogenes *febrile gastroenteritis can also affect healthy people, though many of these cases most probably go unreported.

Adult swine can be infected by *L. monocytogenes *but rarely develop disease [2] and the bacterium is not commonly isolated from swine faeces [3-5]. However, pork meat products have been linked to human infection [6,7] and contamination of the slaughter and processing environment has been traced back to healthy carrier pigs [8]. In ruminants, *L. monocytogenes *can cause neurological disease and abortion, but in general, animals infected are asymptomatic carriers that shed the bacterium in their faeces [2]. Faecal contamination of the farm environment favours animal re-infection and persistence of the pathogen in the production units [9]. In addition, the widespread distribution of *L. monocytogenes *in nature and soil environments is favoured by its ability to grow in a wide range of temperature and pH [10]. This is particularly important in silage production since in many cases the pH reached in the fermentation process is not low enough to prevent growth of *L. monocytogenes *[11]. Therefore, ruminants fed on silage are at higher risk of getting *L. monocytogenes *infection [12].

Hence, animal production units may represent a reservoir for *L. monocytogenes *and source for human infection via faecal contamination of food products since certain *L. monocytogenes *types carried by farm animals have been associated with human infections [13,14]. Though eradication from the farm is highly unlikely due to the ability of *L. monocytogenes *to survive and multiply in many habitats and hosts, transmission and contamination load could probably be reduced through the implementation of adequate intervention strategies. In this context, this study was aimed at determining the prevalence and strain diversity of *L. monocytogenes *in healthy ruminants and swine herds, as a first step before establishing efficient farm-based control measures.

## Results

### Herd and within-herd prevalence

*L. monocytogenes *was isolated from 93 of the 343 herds included in the study (Table 1). *L. monocytogenes *was absent from all 17 porcine herds analysed, but present in cattle (30.6% of beef cattle and 46.3% of dairy cattle herds) and sheep (14.2%). *L. monocytogenes *herd prevalence was significantly (*p *= 10^-5^) higher in cattle than sheep. Among cattle, *L. monocytogenes *was significantly (*p *= 0.0165) more prevalent in dairy than in beef cattle herds (Table 1). In two of the four farms where both sheep and cattle herds were sampled no infection was detected, in the third one *L. monocytogenes *was isolated from cattle but not from sheep, and in another both herds were positive and selected for the *Within-herd prevalence study *(Herds A_S _and A_C _in Table 2). Positivity was scattered throughout the different regions and no clear geographical pattern was observed. No correlation was found between herd and animal census and *L. monocytogenes *prevalence by province or county (*p *> 0.05). However, in the county with the largest census prevalence values were always above the mean.

**Table 1:** Herd prevalence values by animal source

**Animal source**	**Herds analysed**	**Positive Herds**	
		
		**N**	**% (CI)**
Sheep	120	17	14.2 (8.6–19.8)
Beef Cattle	124	38	30.6 (22.8–38.4)
Dairy Cattle	82	38	46.3 (36.0–56.6)
Swine	17	0	0.0

TOTAL	343	93	27.1 (22.7–31.5)

CI, 95% confidence intervals on the mean prevalence.

**Table 2:** Prevalence and typing results of the Within-herd prevalence study

**Herds^a^**	**County^b^**	**n animals analysed**	**n (%) positive animals**	**Serotype^c^**	**PFGE type^d^**
Sheep (A_S_)	1	49	1 (2.0)	4b	LA-04
Sheep (B)	17	48	2 (4.1)	4b	LA-02; LA-24
Sheep (C)	7	50	0 (0.0)	n.a.	n.a.
Sheep (D)	1	50	0 (0.0)	n.a.	n.a.
*TOTAL Sheep*		*197*	*3 (1.5)*		
Dairy cattle (A_C_)	1	46	5 (10.9)	4b (4); 1/2a	LA-08; LA-33; LA-34; LA-35; LA-36
Dairy cattle (E)	3	47	34 (72.3)	4b	LA-07; LA-28; LA-29; LA-30
Dairy cattle (F)	18	50	3 (6.0)	4b (2); 1/2a	LA-05; LA-31; LA-32
Dairy cattle (G)	1	39	2 (5.1)	4b	LA-03; LA-46
*TOTAL Dairy Cattle*		*182*	*44 (24.1)*		
Beef cattle (H)	1	39	3 (7.7)	4b	n.d.

*TOTAL*		*418*	*50 (11.9)*		

^a ^Herds were designated A-H; herds A_S _and A_C _correspond to a sheep and a cattle herd, respectively, that share the same farm premises

^b ^See Fig. 3 for geographical location of counties

^c ^One isolate per positive animal was serotyped, except for herd E for which only 5 isolates were available for serotyping

^d ^PFGE patterns as shown in Fig. 1 n.a., not applicable;

n.d., not done

A total of 418 animals from 9 herds (4 sheep, 4 dairy cattle and 1 beef cattle) were individually analysed for *L. monocytogenes *faecal shedding. Within-herd prevalence for *L. monocytogenes *was higher in dairy cattle (24.1%) than in beef cattle (7.7%) or sheep (1.5%) (Table 2). This difference was also seen in the farm with infected sheep (A_S_: 2.0% shedders) and dairy cattle (A_C_: 10.9%) (Table 2). However, the proportion of shedders varied considerably among herds, especially among dairy cattle (5.1 – 72.3%). Differences were smaller among ovine herds, but in two of them no positive animals where detected. No significant seasonal variation in herd prevalence was observed (*p *< 0.05).

### Distribution and characterisation of *Listeria monocytogenes *isolates

A total of 114 *L. monocytogenes *isolates were serotyped (Table 3), including one isolate from each positive pool (93 isolates from the *Herd prevalence study*) and 21 isolates from the *Within-herd prevalence study *(1–5 isolates per herd from 7 herds, see Table 2). Most of the isolates (84.2%, 96 isolates from 76 herds) were identified as serotype 4b complex. The remaining were serotype 1/2a (13.2%, 15 isolates from 15 herds), 1/2b (1.7%, 2 isolates from 2 herds), and 4c (1 isolate, 0.9%). Serotypes 4b complex and 1/2a were found in sheep and cattle, whereas serotype 1/2b isolates were found in dairy cattle and the 4c isolate in beef cattle. In 2 dairy cattle herds, different serotypes (4b complex and 1/2a) were identified. No association was found between sampling season and serotype.

**Table 3:** Serotype distribution of isolates by animal source

**Animal source**	**Serotype**				**Total**
		
	**1/2a**	**1/2b**	**4b complex^a^**	**4c**	
Sheep	4	-	16	-	20
Beef Cattle	4	-	37	1	42
Dairy Cattle	7	2	43	-	52

TOTAL	15 (13.2%)	2 (1.8%)	96 (84.2%)	1 (0.9%)	114

^a ^serotype 4b and the closely related, albeit rarely encountered serotype 4d and 4e

**Figure 1:**
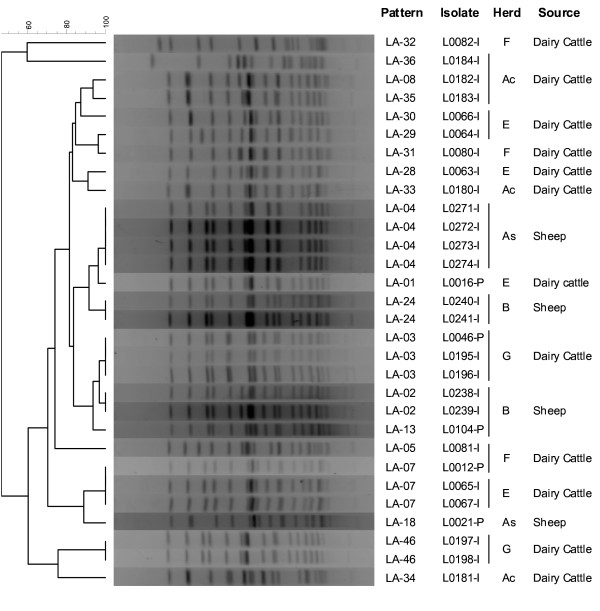
**Dendrogram (UPGMA) of the *Apa*I PFGE patterns of *L. monocytogenes *isolates from 6 different herds (A-G as in Table 2)**. The isolates have been coded according to sample type of origin: P, pool of faeces; I, individual faecal sample.

**Figure 2:**
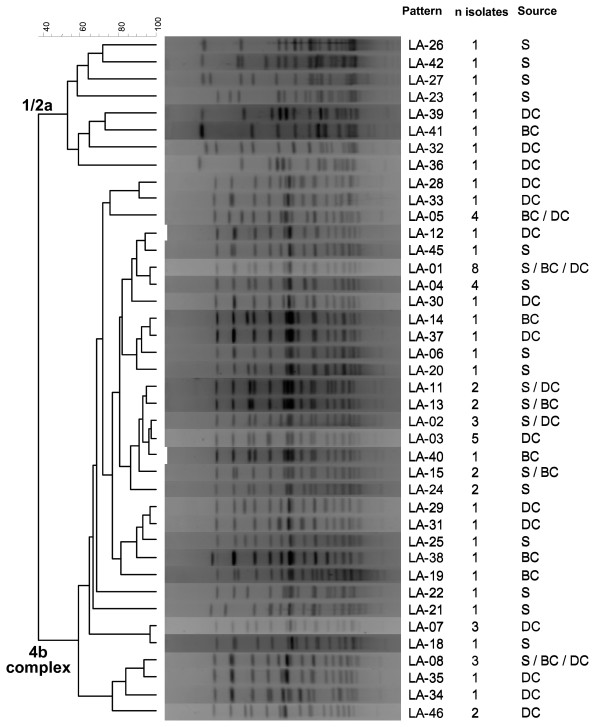
**Dendrogram (UPGMA) of all the different *L. monocytogenes Apa*I PFGE patterns**. Sources were coded as follows: S, sheep; DC, dairy cattle; BC, beef cattle.

*Apa*I PFGE analysis performed on 68 isolates (24 from sheep and 44 from cattle) generated 40 patterns; 28 of them were unique patterns represented by single isolates only, and the remaining 12 included 2–8 isolates. Six patterns where shared by isolates obtained from sheep and cattle. However, isolates obtained from the sheep and cattle herd sharing the same farm premises (herds A_S _and A_C_) were clearly different according to their PFGE types (Table 2, Fig. 1). Serotype 1/2a isolates formed a separated cluster from the 4b complex isolates at similarity levels below 40% (Fig. 2). Diversity was high among 1/2a isolates (8 isolates, 8 patterns below 75% similarity), whereas for 4b isolates, 7 PFGE clusters (each containing 2–6 patterns) were observed at the 90% similarity level. Two of these clusters were represented by 14 and 15 isolates each, isolated from both sheep and cattle. The most prevalent pattern (LA-01, see Fig. 2) was isolated from a sheep herd, 2 beef cattle herds and 2 dairy cattle herds. Another pattern found in the three production systems was LA-08 (Fig. 2). The analysis of 24 ovine isolates from 16 herds generated 19 patterns, whereas in cattle 27 patterns were identified among the 44 isolates obtained from 24 herds. Most of the ruminant isolates analysed by PFGE had been isolated from faecal pools from different herds. However, in 6 herds several isolates originating from individual faecal samples were analysed and in 5 of them nearly all isolates (2–5 isolates/herd) had different patterns (Fig. 1, Table 2), demonstrating that several strains coexisted in the herds. Also interesting was to identify the same pattern in two dairy cattle herds (pattern LA-07 in Herds E & F, Fig. 1) located 63 km apart.

## Discussion

This is the first study carried out in farms from the Basque Country to determine the prevalence of *L. monocytogenes *in healthy animals. Comparison of prevalence results among different studies can be influenced by variation in sampling strategies and season, and differences in detection methods used. In addition, most data on *L. monocytogenes *in ruminants are obtained from cases of clinical listeriosis. Although day-to-day variation in *L. monocytogenes *faecal shedding in dairy cattle has been demonstrated [14], single day sampling can provide an initial snap-shot image of the general situation with regard to the prevalence of *L. monocytogenes *in healthy herds in a region where no such data are available. In this manner, the proportion of faecal shedders observed in this study was similar to that reported by Nightingale et al. [9] among ruminants without clinical symptoms. Likewise, prevalence was higher in cattle (particularly dairy cattle) than sheep. Nightingale et al. [9] showed that contrary to sheep, cattle exposed to *L. monocytogenes *through contaminated silage amplify the pathogen to high levels subsequently increasing faecal shedding, and thus contributing to the maintenance and dispersal of *L. monocytogenes *into the farm environment. A pronounced seasonal variation in faecal shedding has been reported in cattle farms with peak prevalences during the colder months associated to increased silage feeding [15]. Quality of feed provided during the indoor season has also been considered an important risk factor for listeriosis in ruminants [15]. Samples for the *Herd prevalence study *were collected throughout the year, and no seasonal variation in herd prevalence was observed. However, it is noteworthy that the highest within-herd prevalence of *L. monocytogenes *shedders corresponded to a dairy cattle herd sampled in winter (dairy cattle herd E in Table 2). Nevertheless, in the Basque Country, sheep and beef cattle spend most of the year pasture-grazing outdoors, whereas dairy cattle are kept indoors throughout the year under a diet based on silage, which has been reported as a risk for *L. monocytogenes *infection [12]. Stress associated to lactation in dairy cattle might affect susceptibility to *L. monocytogenes *infection [2] and contribute to differences in shedding between beef and dairy cattle. Daily variability in the number of faecal shedders [14] could explain the wide differences in the within-herd prevalence values observed among the four dairy cattle herds, and the absence of shedders in two ovine herds when sampled individually. The higher prevalence of ruminant herds positive for *L. monocytogenes *in areas with larger animal census suggests a possible relation between animal density and infection risk, however, more extensive epidemiological data collection (herds contact at mountain pastures and animal trade) and environmental sampling are needed to confirm this link and identify sources of contamination and infection routes.

Swine production in the Basque Country is not extensive and therefore, the number of swine herds analysed in this study was limited. However, *L. monocytogenes *was never detected in the pig herds analysed. Although *L. monocytogenes *occurs frequently in pork products, it is rarely isolated from swine faeces [3-5]. Higher infection rates are detected in skin swabs [16] or tonsils [3], and the prevalence of *L. monocytogenes *in swine generally increases from the farm to the manufacturing plants. Hence, the main source for contamination with *L. monocytogenes *appears to be the slaughter and processing environment where *L. monocytogenes *can survive for long periods [17]. Listeriosis is frequently traced to ready-to-eat (RTE) meat products, regardless of meat animal source, and some delicatessen RTE pork products have been involved in listeriosis outbreaks [6,7]. Conversely, bovine or ovine meat products are rarely associated to human listeriosis, but ruminant healthy carriers may shed *Listeria *in faeces contaminating pastures or vegetables [18], surface waters [19] and milk [20]. In a study carried out in Navarra (Northern Spain) on food samples from different industries and markets [21], the incidence of *L. monocytogenes *in raw minced beef and pork meat was 34.9%, 5.4% in raw milk samples (6.8% in cattle milk and 3.0% in sheep milk), and 1.0% in soft cheese. Nevertheless, unpasteurised dairy products represent the major problem regarding human infection.

Strain typing can help to identify sources of infection and routes of transmission and in this sense, is commonly used for disease tracking in human infections. *L. monocytogenes *comprises a diversity of strains classified into 13 different serotypes, however, only three (1/2a, 1/2b and 4b) are commonly associated with human listeriosis [21,22]. Food-stuffs are mainly contaminated by serogroup 1/2 isolates, whereas most human clinical isolates belong to serotype 4b [21,22], and among these, a small subgroup with unique gene clusters represent the two major epidemic-associated clonal groups [23,24]. In this study, serotype 4b complex was the most common in ruminants (84.2% of isolates and 81.7% of positive herds), followed by serotype 1/2a (13.2% of isolates and 14.0% of positive herds). Since each pool of faecal material can contain several *L. monocytogenes *strains with different serotype, these values cannot be interpreted as serotype prevalences but clearly indicate the predominance of serotype 4b in healthy ruminants from the Basque Country. As part of the USA National Animal Health Monitoring System Dairy 2002 survey, Van Kessel et al. [25] found a varied distribution of serotypes among *L. monocytogenes *isolated from cattle bulk tank milk in different regions of the country, with serotype 4b predominating in the Southeast and serogroup 1/2 elsewhere [25]. Serogroup 1/2 was predominant in beef and pork raw minced meat samples from different industries and markets in Navarra (Northern Spain); in cattle milk samples, serogroups 1/2 and 4 were similarly represented (47.8 and 39.1%, respectively), but in sheep milk 83.3% of the isolates were serotype 4b [21]. Conversely, in this study we detected serotype 1/2a at higher proportion in sheep than in cattle. Reporting of human listeriosis cases is compulsory in Spain and Microbiology laboratories at the hospital setting report isolations weekly. A 16-year survey carried out from 1990 to 2006 in one of the largest hospitals in the Basque Country identified 60 human clinical cases, with serotype 4b representing 78% of the cases [26], similar to other Spanish regions [27,28]. Serotype 1/2b, only represented by two dairy cattle isolates in this study, was second (14%) among local human cases, whereas serotype 1/2a, second among animal samples, was the less common among human cases (6.8%) in the Basque Country [26]. In this context, serotyping is, however, of limited discriminatory value, and techniques like PFGE provide enhanced discrimination for outbreak investigations and surveillance purposes. Restriction analysis with *Apa*I performed on 68 isolates generated 40 patterns, and provided serotype-specific PFGE patterns that clearly separated 1/2a isolates from 4b isolates, and differentiated strains within serotypes. These results confirmed previously established relationships between serotype and PFGE patterns [22], and revealed that the *L. monocytogenes *population in Basque farms is genetically highly diverse. In general, isolates from different herds were very different, but occasionally identical or similar patterns were observed in different herds. Contact at communal mountain pastures could be an occasion for strain exchange among sheep and beef cattle herds. However, since dairy cattle are confined indoors, the identification of certain patterns in the three production systems or in dairy cattle herds distantly located, suggest other sources of infection. On the other hand, strains differing in more than seven bands, and therefore of limited genetic relatedness [29], were also identified within each herd suggesting multiple sources of contamination.

## Conclusion

Listeriosis results in losses to the agricultural economy due to illness and increased infertility and abortion rates, but losses also occur when consumer confidence is undermined as a consequence of food-borne infections. Food safety programs that cover all aspects of food production (from farm to fork) are needed to provide a safe food supply and prevent foodborne illnesses. Identification of on-farm reservoirs is a pre-requisite for the implementation of farm-specific pathogen reduction programs. In this sense, this study showed a high prevalence of *L. monocytogenes *in ruminant herds compared to swine, suggesting that such herds may represent an important reservoir for *L. monocytogenes *in the Basque Country. The wide distribution and variability in *L. monocytogenes *shed within and among ruminant herds highlighted the complexity of pathogen control at the farm level. The ubiquitous nature of this pathogen hampers its total removal from the farm environment, but a reduction of the intestinal carriage rate in livestock herds would contribute to reduce the contamination pressure at the slaughterhouse and dairy production. In any case, since the relatively high prevalence of *L. monocytogenes *in ruminant herds does not correlate with the low incidence of human infections in the Basque Country, it can be speculated that control measures to avoid contamination of final food products are being efficient or, possibly, these could be animal-adapted strains with reduced ability to cause human infections. Continuous monitoring schemes and surveillance programs are needed to evaluate trends in the occurrence of *L. monocytogenes *in livestock and to prevent food contamination.

## Methods

### Sampling design

**Figure 3:**
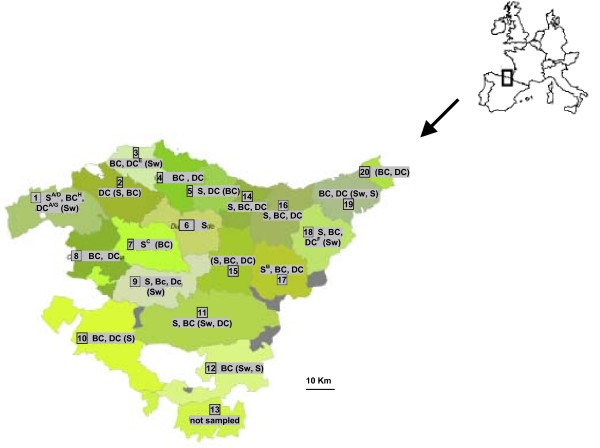
**Map of the Basque Country**. Counties are labelled with numbers (1–7 counties in Bizkaia; 8–13 in Araba; 14–20 in Gipuzkoa). Herd animal species sampled in each county are indicated (S, sheep; DC, dairy cattle; BC, beef cattle; Sw, swine), showing in brackets herd species that tested negative. Farms sampled for the Within-herd prevalence study are indicated in superscript.

Healthy swine, cattle (beef and dairy) and dairy sheep herds were sampled to estimate *L. monocytogenes *prevalence in farms from the Basque Country, a 7,200-km^2 ^region located in Atlantic northern Spain that is divided into three provinces, each of them formed by several counties: Bizkaia (counties 1–7 in Fig. 3), Araba (8–13) and Gipuzkoa (14–20). Swine production in the Basque Country is not very extensive (*ca*. 40,000 animals) and is based on indoor confinement systems mainly located in the southern counties. Approximately 40% of the population corresponds to suckling pigs that are fed elsewhere, whereas sows constitute 20% of the animals. The ovine population includes *ca*. 322,000 sheep of Latxa dairy breed that are commonly housed in winter and during milking (one lambing per year in November-March), but have access to summer communal mountain pastures. The cattle population includes *c.a*. 170,000 animals of which about 60% are beef cattle and the remaining 40% dairy cattle. Only occasionally do cattle and sheep herds share farm premises.  In general, sheep and beef cattle spend most of the year pasture-grazing outdoors and during summer months they usually share mountain pastures, generally within the county but occasionally crossing county boundaries (see Fig. 3); conversely, dairy cattle are kept indoors throughout the year under a diet based on silage.

Two different sampling strategies were followed: one designed to determine the number of herds positive for *L. monocytogenes *(*Herd prevalence*), and another to establish the proportion of individuals shedding the organism (*Within-herd prevalence*) within a selected number of herds identified as positive in the first approach. To estimate herd prevalence, a statistically adequate sample size was calculated on the basis of census provided by the Department of Agriculture of the Basque Country as previously described [30]. Thus, for the *Herd prevalence study*, a total of 343 herds (17 swine, 120 dairy sheep, 124 beef and 82 dairy cattle) distributed through the different regions were visited once, and faecal samples were collected from the rectum of 30 animals per herd and screened in one pool. Distance between farms ranged between a few metres to 135 kilometres and in four farms both sheep and cattle herds were sampled. *Within-herd prevalence *was established by individually analysing a maximum of 50 animals from a selection of 9 pool-positive herds (4 sheep, 1 beef and 4 dairy cattle) accounting for a total of 418 animals. Samples were collected by official veterinarians from the Diputaciones Forales from October 2003 to May 2005 and cooled samples were sent to the laboratory on the day of collection.

### Isolation and identification of *L. monocytogenes*

Isolation and identification of *L. monocytogenes *was carried out as previously described [31]. Briefly, 25 g of pooled (*Herd prevalence*) rectal faecal samples or 1 g of individual (*Within-herd prevalence*) faeces were diluted 1/10 in Half-Fraser broth (bioMérieux, Marcy-l'Etoile, France), homogenized and incubated for 22 ± 1 h at 30°C for enrichment. One ml of the incubated suspension was transferred to 10 ml of Fraser broth and incubated as above. Suspensions were then screened for the presence of *L. monocytogenes *using VIDAS *Listeria monocytogenes *II test kit (bioMérieux) for automated immunoenzymatic detection. Positive samples were subcultured from the remaining Fraser broth onto a selective and differential agar (Agar Listeria according to Ottaviani and Agosti, ALOA) (AES Laboratories, Combourg, France), and *L. monocytogenes*-presumptive colonies were biochemically identified with a commercial API *Listeria *system (bioMérieux).

### *L. monocytogenes *serotyping and pulsed-field gel electrophoresis (PFGE) analysis

Serotyping was performed through examination of group-specific Listeria O and H antigens [32] by slide agglutination using commercially prepared antisera (*Listeria *antiserum Seiken Kit; Denka Seiken Co., Tokyo, Japan) according to the manufacturer's instructions. Serotype 4b and the closely related, albeit rarely encountered serotypes 4d and 4e, could not always be discriminated by the technique and were designated serotype 4b complex.

For PFGE analysis, pure cultures obtained from single *L. monocytogenes *colonies were suspended into 3 ml of TE buffer (10 mM Tris, 1 mM EDTA, pH 8.0) and adjusted to McFarland standard 4–5 using a densitometer (Densimat, bioMérieux) and blocks were prepared and digested with 200 units of *Apa*I following the PulseNet standard laboratory operating procedure for *L. monocytogenes *PFGE . Fragments were separated by electrophoresis in a CHEF-DRII system (BioRad) at a constant temperature of 14°C during 5 h using an initial switch time of 15 s and a final switch time of 35 s and a further 15 h with switch times of 2–20 s. Gels were normalised by alignment with the Lambda Ladder PFGE size standard (New England Biolabs, MA, USA) and *Salmonella *Braenderup control strain H9812 digested with *Xba*I [33]. Gel images were captured using a Fluor-S™ MultiImager (BioRad) and patterns were compared by GelCompar^® ^II (BioNumerics, Applied-Maths, Kortrijk, Belgium). Similarities between the profiles based on band positions were derived by using the Dice correlation coefficient with maximium position tolerance of 1% and 0.2% optimisation and dendrograms were constructed by the unweighted pair group method (UPGMA). Patterns differing by at least one band (number and/or sizes) were considered different and a new code was assigned.

### Statistical analysis

A chi-squared (*X*^2^) test was used to compare the herd prevalence of *L. monocytogenes *from different sources. Significance of the association between sampling season (colder months *vs*. warmer months) and *L. monocytogenes *isolation and with the serotype were evaluated by Fisher exact probability test. Spearman non-parametric correlation analysis was used to investigate associations between herd and animal population and estimated prevalence. All statistical tests were performed using the SAS statistical package (Version 9.1). *p *values less than 0.05 were considered significant. The 95% confidence intervals on the herd prevalence were calculated using Win Episcope 2.0  for the population size (census provided by the Department of Agriculture of the Basque Country), the sample size (number of herds sampled) and the observed prevalence.

## Authors' contributions

JIE participated in the microbiological analysis and molecular typing. BO performed the microbiological analysis and participated in drafting of the manuscript. GA participated in the design of the study and critically revised the publication. RAJ participated in the design of the study and performed the statistical analysis. AH participated in the design and coordination of the study, analysed the data and drafted the manuscript. All authors read and approved the final manuscript.
